# Layperson’s Preference Regarding Orientation of Transverse Occlusal Plane from the Frontal Perspective in Fabrication of a Complete Denture: A Cross-sectional Study

**DOI:** 10.7759/cureus.6650

**Published:** 2020-01-14

**Authors:** Elias M K, Sriharsha Pudi, Tirumala Ravali C, Rajasekhar Reddy Gade, Bhavan Chand Yemineni

**Affiliations:** 1 Prosthodontics, Care Dental Clinic, Eloor, IND; 2 Prosthodontics and Crown & Bridges, MNR Dental College and Hospital, Hyderabad, IND; 3 Oral Medicine and Radiology, Govt Dental College and Hospital, Hyderabad, IND; 4 Prosthodontics and Crown & Bridges, St. Joseph Dental College and Hospital, Eluru, IND; 5 Dental and Oral Surgery, Alluri Sitarama Raju Academy Of Medical Sciences (ASRAM) College and Hospital, Eluru, IND

**Keywords:** complete denture, canting, dental esthetics, occlusal plane

## Abstract

Background and aim: Assessment of dentofacial asymmetries and other discrepancies that can affect the horizontal reference lines should be considered initially as a part of the esthetic diagnosis. Some variations of facial asymmetry are not considered an esthetic liability. So the aim of the present study is to determine a layperson’s preference regarding transverse occlusal plane (TOP) orientation in fabrication of a complete denture.

Materials and methods: A total of 100 subjects who are edentulous and have enrolled for complete denture fabrication were selected. Photograph of the patient’s face was obtained from the frontal perspective on the day of try-in. The image obtained was edited to orient the occlusal plane in three different cants, zero degree, two degrees, and four degrees to the inter-pupillary line (IL) and presented to the patient. The obtained results were statistically analyzed.

Results: The observed data was analyzed using Friedman’s test and Wilcoxon test was used for comparing ordinal data between groups. There was a statistically significant difference in acceptance depending on angulation, χ2(2) = 183.2, p = 0.0001.

Conclusion: A cant of two degrees is not perceived by the subject but some subjects’ preference of occlusal plane may be altered according to the commissural canting which cannot be incorporated in complete denture fabrication.

## Introduction

The transverse occlusal plane (TOP) when viewed from a frontal perspective should be parallel to the facial horizontal reference lines such as the inter-pupillary line (IL) and the commissure line (CL) to maintain facial harmony. Lack of parallelism among the TOP, CL, and IL can be attributed to different causes. The presence of an elevated labial commissure at rest or alar base on one side is often an indication of vertical skeletal asymmetry. Conditions for each patient should be individually diagnosed. Restorative treatment alone can also be a solution depending on a particular situation and the patient’s individual needs. Assessment of dentofacial asymmetries and other discrepancies that can affect the horizontal reference lines should be considered initially as a part of esthetic diagnosis.

Laypeople prefer some degree of TOP inclination in patients who present a lip CL cant. The acceptable degree of canting should be assessed on an individual patient basis. Therefore, the purpose of the study is to determine a layperson’s preference regarding TOP orientation in fabrication of a complete denture.

## Materials and methods

**Figure 1:**
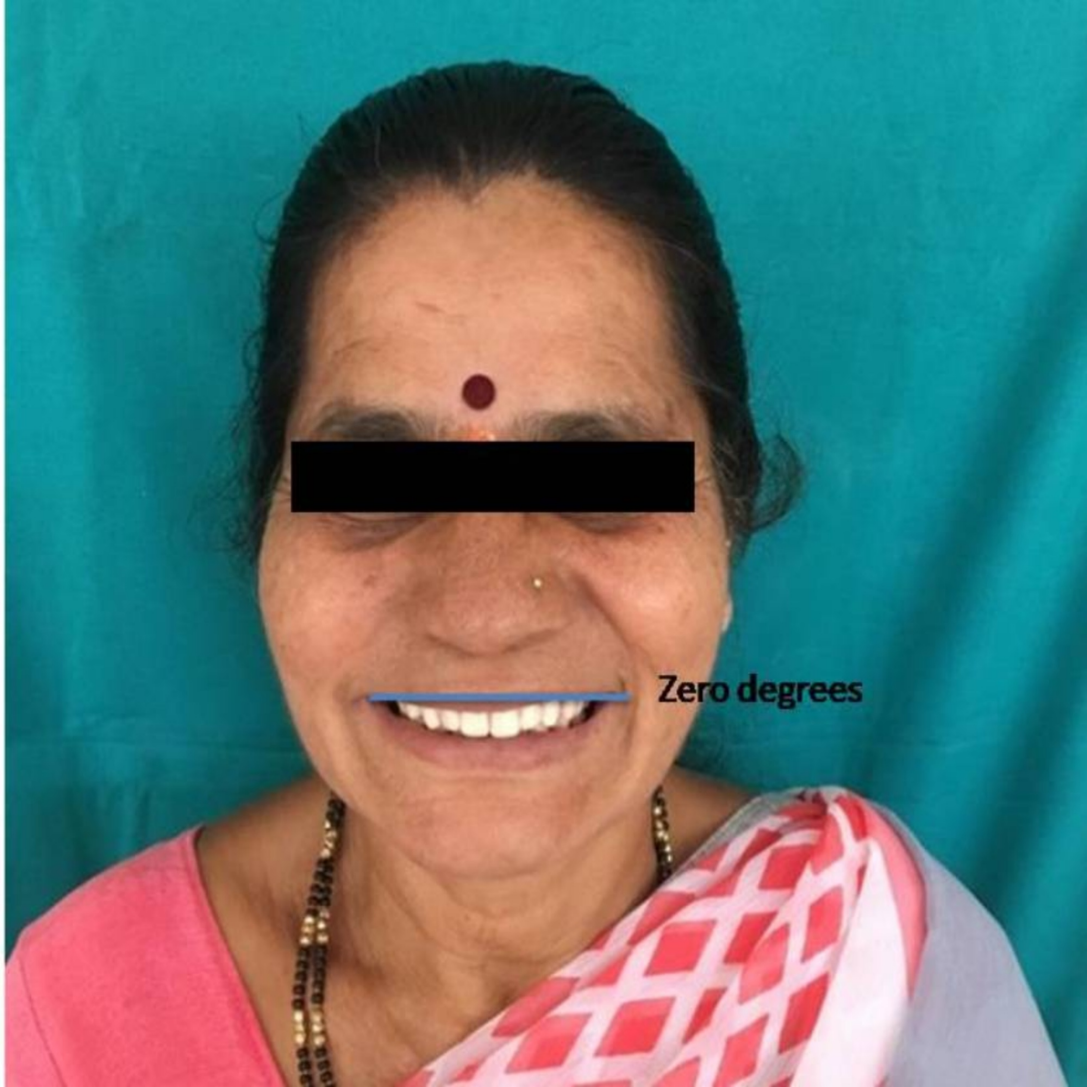
TOP mean between angle of inter-pupillary plane equivalent to zero degrees. TOP, transverse occlusal plane

**Figure 2:**
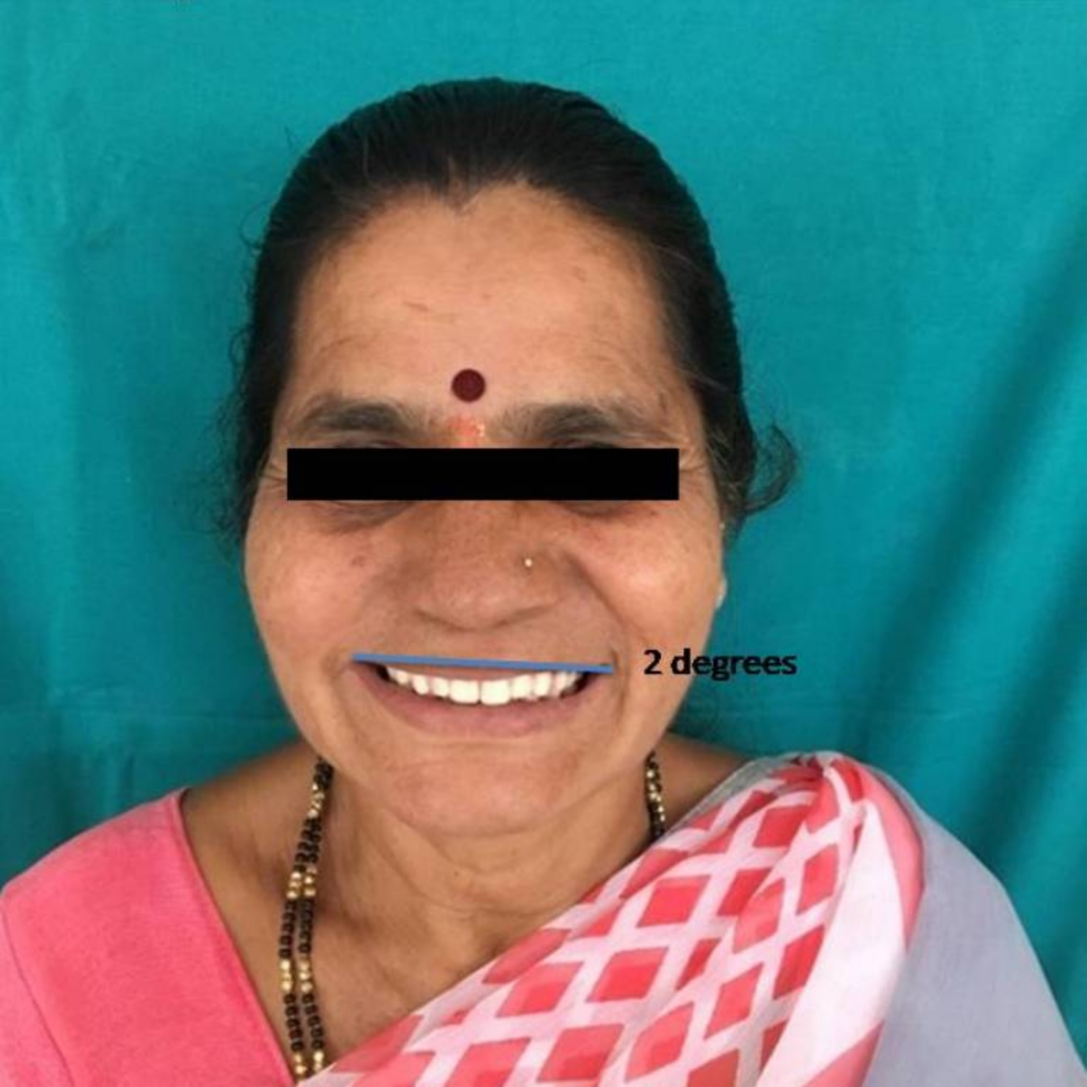
TOP mean between angle of inter-pupillary plane equivalent to two degrees. TOP, transverse occlusal plane

**Figure 3:**
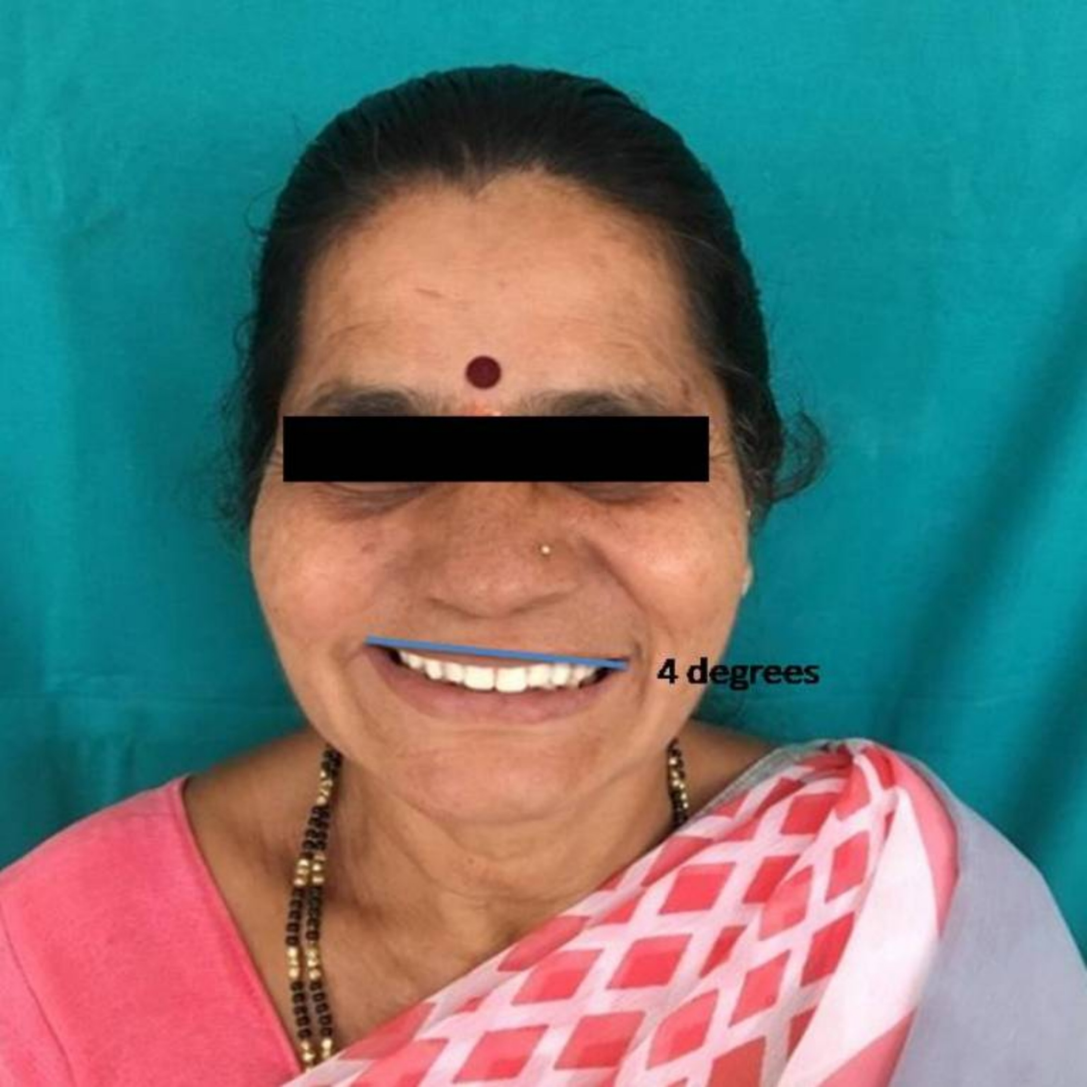
TOP mean between angle of inter-pupillary plane equivalent to four degrees. TOP, transverse occlusal plane

The study design was a double-blinded observational trial. The study plan was approved by the institutional ethical committee. Participants were explained about the nature of the study and written informed consent was obtained. A total of 100 subjects who are edentulous and have enrolled for complete denture fabrication were selected from the outpatients in the Department of Prosthodontics and Crown & Bridge, JSS Dental College and Hospital, Mysuru. Conventional complete denture fabrication procedures were followed to fabricate upper and lower complete denture and on the day of try-in of the complete denture, photograph of the patient’s face was obtained from the frontal perspective. The image obtained was edited to orient the occlusal plane in three different cants, zero degrees (Figure 1), two degrees (Figure 2), and four degrees (Figure 3) to the IL and presented to the patient. The patient’s perception of the different images was recorded. After the data collection, the results were tabulated and statistically analyzed.

## Results

The data were analyzed using the statistical package for social sciences (SPSS) for Windows 26.0 (IBM SPSS, Armonk, NY). The descriptive data show mean, standard deviation (SD) and were used for comparison between the groups. The observed data were analyzed using Friedman’s test and Wilcoxon test was used for comparing ordinal data between groups. Confidence intervals were set at 95% and values of p < 0.05 were interpreted as statistically significant. There was a statistically significant difference in acceptance depending on angulation, χ2(2) = 183,2, p = 0.0001 (Table 1). Posthoc analysis with Wilcoxon signed-rank tests was conducted with a Bonferroni correction applied, resulting in a significance level set at p < 0.017. There were significant differences between different angulations with two-degree angulation receiving high acceptance followed by zero- and four-degree angulations (Table 2).

**Table 1:** Related samples Friedman’s test. *statistically significant

	N	Percentiles			Mean rank	χ^2^	df	p value
		25th	50th (Median)	75th				
Zero degree	100	3.00	3.00	3.00	2.91	183.2	2	0.0001*
Two degree	100	2.00	2.00	2.00	2.07			
Four degree	100	1.00	1.00	1.00	1.03			

**Table 2:** Wilcoxon signed ranks test. a. Based on positive ranks b. Based on negative ranks *statistically significant

	Four degree – two degree	Zero degree – two degree	Four degree – zero degree
Z	-9.715^a^	-8.814^b^	-9.569^a^
p value	0.0001*	0.0001*	0.0001*

## Discussion

According to Glossary of Prosthodontic Terms 9 (GPT 9), an occlusal plane is defined as the average plane established by the incisal and occlusal surfaces of the teeth; it is not a plane but represents the planar mean of the curvature of the surfaces [1]. The position of the occlusal plane in denture wearers should be as close as possible to the plane which was previously occupied by the natural teeth. Such position of the occlusal plane provides a normal function of the tongue and cheek muscles, thus enhancing the denture stability.

The faulty orientation of the occlusal plane will jeopardize interaction between tongue and buccinator muscles. Where the occlusal plane is too high, the tongue cannot rest on the lingual cusps of the lower denture and thus prevents its displacement. It also forces the tongue into a new position that is higher than its normal position causing the floor of the mouth to raise and create undue pressure on the border of the lingual flange, resulting in partial loss of border seal. There is also a tendency for the accumulation of food in the buccal and lingual sulci. An occlusal plane that is too low could lead to tongue and cheek biting [2].

In the anterior region, the vertical height of the occlusal plane is governed by esthetic requirements and less frequently by functional demands [3]. The anterior maxillary occlusal plane may be determined by lip relationships at rest and when smiling. Speech also provides for positional accuracy [4]. When viewed from the front, the occlusal plane should be parallel to the IL [5].

In the present study frontal photograph of the subject was obtained during try-in and was edited using Adobe Photoshop. Three images of the subject with a modified occlusal plane were presented to the subject. Subject perception of the occlusal plane and his/her preference of cant were recorded.

The image manipulated to create a two-degree occlusal cant was esthetically acceptable for 87 subjects. These results coincide with other research that has observed that occlusal canting is not perceived by laypersons unless it exceeds two or three degrees. Kokich et al. [6] found that laypersons did not detect this type of asymmetry unless it reached a four-degree inclination.

Padwa and Kaiser [7] have shown that occlusal canting greater than four degress is detected clinically with a frequency of over 90% by both professionals (trained in this field of observation) and laypersons (untrained in this field). However, Ker et al. [8] observed that laypersons found occlusal canting of up to four degrees acceptable and a third of them found this acceptable up to a maximum of six degrees.

Olivares et al. [9] suggested that the profession of the evaluators affected the evaluation of smile esthetics when a canted occlusal plane was present. He evaluated the perception of a group of orthodontists, general practitioners, and laypersons for evaluating the TOP. The present study result is in line with this study regarding the layperson’s perception of zero-degree and two-degree occlusal cant.

Silva et al. [10] found that an occlusal cant of less than three degrees is not perceived by layperson when viewed from the frontal plane which is in line with the present study. Silva et al. [11] concluded that layperson prefers the occlusal cant in line with the cant of the CL rather than the IL which will explain the subject’s acceptance of six degrees of cant in 13 samples.

## Conclusions

Rehabilitation of completely edentulous individual is a challenging job for a restorative dentist when the esthetic anticipation of the patient is high. A cant of two degrees is not perceived by the subject but some subject’s preference of occlusal plane may be altered according to the commissural canting which cannot be incorporated in complete denture fabrication. So it is the responsibility of the restorative dentist to educate the patient regarding the same in cases of extreme canting to fabricate a denture which has a balance between esthetics and function.
